# Traveling Theta Waves and the Hippocampal Phase Code

**DOI:** 10.1038/s41598-017-08053-3

**Published:** 2017-08-09

**Authors:** Christian Leibold, Mauro M. Monsalve-Mercado

**Affiliations:** 1 Department Biology II, Ludwig-Maximilians-Universität München, Munich, Germany; 2 Bernstein Center for Computational Neuroscience Munich, Munich, Germany

## Abstract

Hippocampal place fields form a neuronal map of the spatial environment. In addition, the distance between two place field centers is proportional to the firing phase difference of two place cells with respect to the local theta rhythm. This consistency between spatial distance and theta phase is generally assumed to result from hippocampal phase precession: The firing phase of a place cell decreases with distance traveled in the place field. The rate of phase precession depends on place field width such that the phase range covered in a traversal of a place field is independent of field width. Width-dependent precession rates, however, generally disrupt the consistency between distance and phase differences. In this paper we provide a mathematical theory suggesting that this consistency can only be secured for different place field widths if phase precession starts at a width-dependent phase offset. These offsets are in accordance with the experimentally observed theta wave traveling from the dorsal to the ventral pole of the hippocampus. Furthermore the theory predicts that sequences of place cells with different widths should be ordered according to the end of the place field. The results also hold for considerably nonlinear phase precession profiles.

## Introduction

Hippocampal place cells fire action potentials (spikes) in only few locations of an environment forming a neuronal map of space^1^. The spike times of place cells are coordinated with the extracellular field potential oscillations in the theta range (4–12 Hz). In experiments with rodents, it was shown that place cells spike late in the theta cycle when the animal enters a place field, and subsequently precess to early theta phases during traversal of the place field^2–5^. As a result of this phase precession, the spike times of a population of place cells arrange as theta sequences that encode trajectories in space^2–4, 6–11^: Within each theta cycle cells fire first (at early phases) whose place fields are almost completely traversed, whereas cells fire latest whose place fields have just been entered, and, thus, theta sequences are generally considered as time-compressed representations of the spatial trajectories during behavior. Moreover, theta sequences imply causal pairwise correlations on the theta time scale that can trigger spike-timing-dependent plasticity rules^12–15^ and thereby imprint the memories of spatial trajectories into the synaptic matrix of the hippocampal network^4, 7, 16^.

**Figure 1:**
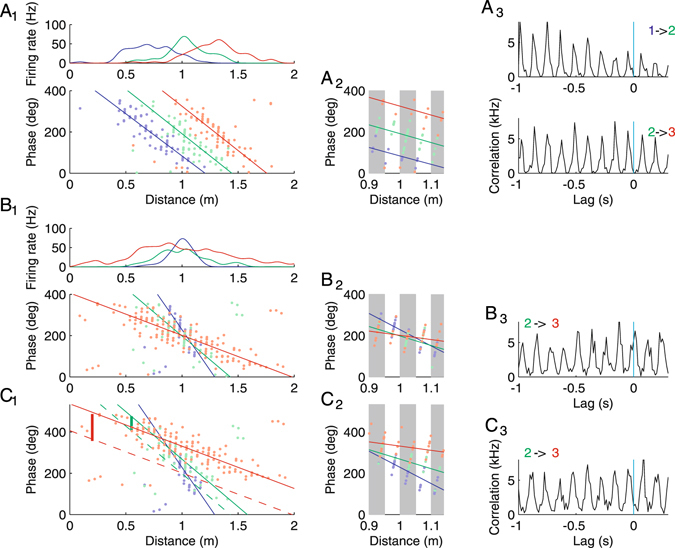
Phase precession and pairwise correlations in simulated place field activity. (**A**_**1**_) Spike phases and positions of three cells (colors) with same width and different centers. Top panel depicts firing rates as a function of position. Bottom depicts phase of spikes as a function of position. Solid lines are obtained from circular linear fits to the dots^34^. (**A**_**2**_) Close up of phase plot from A_1_ at the place field center illustrating that in each theta cycle (grey and white columns) the temporal sequence of spikes (vertically increasing phase patterns) corresponds to ordered place fields: 1 before 2 before 3. (**A**_**3**_) Crosscorrelation functions exhibit systematic peak shifts on the theta time scale encoding the difference of the place field centers. The negative peak lags correspond to cell 1 firing before cell 2 and cell 2 firing before cell 3 as indicated by the labels (1 → 2, 2 → 3). (**B**_**1–3**_) Same as in A for three place cells with different widths and same center. Cells 2 and 3 fire at the same time on average. (**C**_**1–3**_) Data from B with a cell specific phase shift: The larger the field the more the phases are shifted upwards. Dashed lines are the linear fits from B_1_. Thick solid vertical lines indicate the theta phase offsets relative to the local theta oscillation of the blue cell: Theta oscillation is increasingly delayed for the green and the red cell. For details on the numerics see Methods.

An implicit assumption underlying most of the ideas about decoding theta sequences and their implications for learning is that the rate of phase precession is equal in all neurons and thus accounts for the consistency between contiguous space and the circular theta phase (Fig. 1A); i.e. the phase difference of two neurons is directly proportional to the spatial distance between the positions encoded by the two neurons. However, place cells show different rates of phase precession depending on their place field width^3, 8^: Cells with broad place fields precess slower than those with narrow place fields such that the phase range is constant independent of place field width. Owing to the different phase precession rates, spike timing relations between pairs of cells disorganize and cell pairs may swap their order of firing during the course of place field traversal (Fig. 1B).

A further challenge to the assumption that spatial displacements of place fields are consistently encoded as phase differences (called the consistency assumption in what follows) is that the theta oscillation does not constitute a globally synchronized hippocampus-wide oscillation but rather exhibits traveling-wave-like behavior along the dorso-ventral axis^17–20^ resulting in a systematic phase shift of the local theta oscillations across the hippocampus. Thus the spikes of more ventral cells, which have larger place fields^21–25^ (and precess slowly), generally occur later in time than the spikes of dorsal cells, which have smaller place fields (and precess fast) despite their *local* theta phases might be identical.

Owing to the field-width dependent phase precession rate and the traveling theta wave it is unclear whether the consistency assumption holds across the whole hippocampus, or whether it is confined to only a limited region on the dorso-ventral axis. Since, however, the recurrent connectivity of the hippocampal CA3 network extends across at least 2/3 of the dorso-ventral axis^26^, it is conceivable that the consistency assumption must hold across the whole hippocampus, if the temporal contingency of place field spiking was a fundamental organizational principle of the hippocampal code. So far there is some evidence that parts of the trajectories can indeed be decoded from theta sequences across large parts of the dorso-ventral extent of the hippocampus^20^, however, a consistent theoretical framework is missing.

In this paper we provide a theoretical analysis of the consistency assumption taking into account variable place-field-width-dependent rates of phase precession. Our analysis shows that the consistency assumption can indeed be secured in a traveling wave framework if cells with broader place fields phase precess with respect to a local theta rhythm that is delayed compared to the local theta rhythm for cells with smaller place fields (Fig. 1C). From an optimality argument, we will derive that the maximal phase shift between the hippocampal theta oscillations in the dorsal and the ventral hippocampus should be about 180° as was found experimentally^18, 19^.

## Methods

### Numerical Simulations

For Fig. 1 we simulated place field activity as inhomogeneous Poisson processes with density

1 $$\lambda (t)\propto \exp [-\frac{{(vt-{x}_{0})}^{2}}{2{\sigma }^{2}}]\,{[1+{\rm{c}}{\rm{o}}{\rm{s}}({\omega }_{c}(t-{x}_{0}/v))]}^{4}$$

were *v* denotes running speed, *t* is time, *x*_0_ the place field center, *σ* specifies place field width, and *ω*_*c*_ = 2*π*/*T*_*c*_ is the oscillation frequency of the individual neurons. In all simulations we assumed a linear path with constant speed *v* = 40 cm/s. The oscillation period of a cell was width dependent to ensure a width independent phase range, ${T}_{c}={T}_{\theta }\,(1-0.06\frac{{\sigma }_{0}}{\sigma })$, with *σ*_0_ = 20 cm and the theta period *T*_*θ*_ = 1/8 s.

## Results

### Model

**Figure 2:**
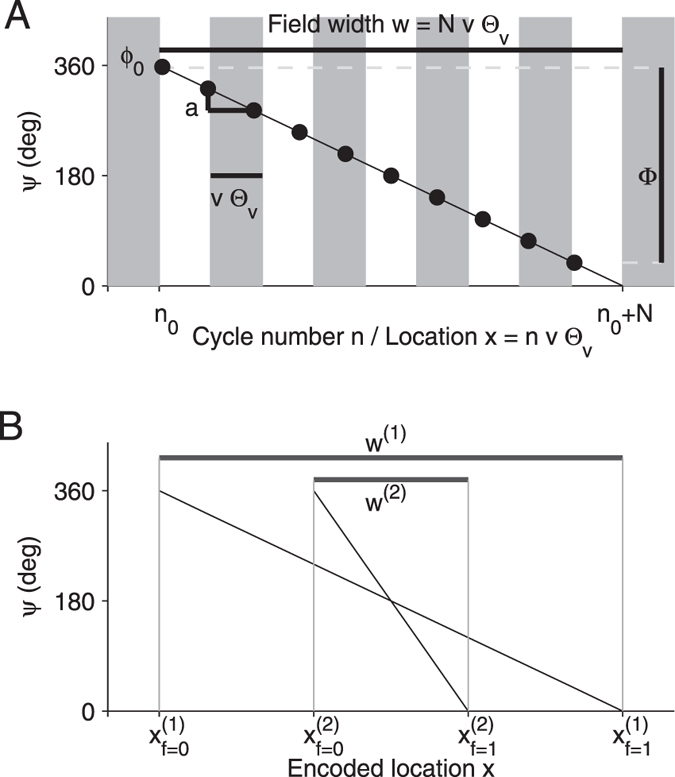
Nomenclature. (**A**) Spike phases *ψ* (black circles) decrease by *a* in each theta cycle (alternating grey and white patches). A field starts at theta cycle *n*_0_ and ends at cycle *n*_0_ + *N* (here *N* = 9). The start phase is denoted *ϕ*_0_, the total phase range is denoted by Φ < 0. The width *w* of the field is a result of the number *N* of theta cycles needed to cover the phase range. The spatial distance an animal covers in one theta cycle equals *v*Θ_*v*_, in which *v* denotes running speed and Θ_*v*_ denotes the period length of a theta cycle in time. (**B**) To define a ordered sequence of overlapping place fields of different widths, we introduce the parameter *f* (fraction in the field), which identifies the field’s location in the sequence. For *f* = 0 (beginning of the field) the proposed sequential ordering of the two fields is 1 → 2, for *f* = 1 (end of the field) the ordering is 2 → 1.

We assume that within a place field the theta phase *ψ* of the spikes decreases linearly by a constant amount *a* per cycle (see Fig. 2A for illustration). If the animal enters the place field of cell *i* at theta cycle ${n}_{0}^{(i)}$, the phase ${\psi }_{n}^{(i)}$ of neuron *i* at cycle *n* is thus described by

2 $${\psi }_{n}^{(i)}={\varphi }_{0}^{(i)}-a(v,{w}^{(i)})(n-{n}_{0}^{(i)}),n\ge {n}_{0}^{(i)}.$$

The phase offset ${\varphi }_{0}^{(i)}$ denotes the starting phase at the entrance of the field. Most importantly, this offset is cell specific (indicated by *i*) and thus the local theta rhythm is not introducing a hippocampus-wide temporal reference. Later on, we will relate ${\varphi }_{0}^{(i)}$ to the hippocampus-wide temporal reference frame introduced by the travelling theta wave. The slope parameter *a* > 0 in equation (2) depends on both the running speed *v* of the animal and the width *w* ^(*i*)^ of the place field, defined as the spatial distance between the occurrence of the first and the last theta cycle. The slope is such that the phase range Φ that is covered while fully crossing a place field during *N* ^(*i*)^ theta cycles is independent of speed^27, 28^ and width^8, 27, 29^, and identical for all cells, i.e.,

3 $${\psi }_{{n}_{0}^{(i)}+{N}^{(i)}}^{(i)}-{\psi }_{{n}_{0}^{(i)}}^{(i)}={\rm{\Phi }}\Rightarrow -a(v,{w}^{(i)}){N}^{(i)}={\rm{\Phi }}.$$

Experimental reports restrict the phase range to values below 2*π* ^5, 30, 31^, and therefore we can treat the phases *ψ* as linear variables without the risk of potential ambiguities.

The place field width *w* ^(*i*)^ can be expressed in terms of the speed-dependent theta period Θ_*v*_ and the running speed *v* according to

4 $${w}^{(i)}=v{{\rm{\Theta }}}_{v}{N}^{(i)}$$

and thus the slope parameter equals

5 $$a(v,{w}^{(i)})=\frac{-{\rm{\Phi }}v{{\rm{\Theta }}}_{v}}{{w}^{(i)}}.$$

### Coding Assumptions

Place field activity encodes a spatial position, however, it is not a priori clear what exact position this should be since place fields are extended in space. For the rate code, the place field is generally interpreted in a probabilistic way in that the firing rate is seen as a correlate for the probability of being at a certain position. This argument, however, does not pertain to a timing code. We thus have to make additional assumptions about the encoding of space by the theta phase. In particular, thinking about sequences of place cells we have to deal with the problem of which of the two place fields comes earlier in the sequence. Since place fields have different widths, this sequence will be generally different depending on whether we order the fields according to their beginning, their center, or their end. In the following we assume that the timing of a place cell spike encodes the distance to the sequence position

6 $${x}^{(i)}:=({n}_{0}^{(i)}+{n}_{x}^{(i)})v\,{{\rm{\Theta }}}_{v},{n}_{x}^{(i)}=f{N}^{(i)},$$

i.e., the distance to the position that corresponds to the fraction *f* of the run through the place field. Choosing *f* = 0 would mean that a spike encodes the distance from the beginning of the place field, *f* = 1/2 would mean that a spike encodes the distance to middle of the field and *f* = 1 indicates the distance to the end of the field (see Fig. 2B for illustration).

### Phase Difference

Equations (4) and (6) let us relate the starting cycle ${n}_{0}^{(i)}$ to the reference position *x* ^(*i*)^ via

7 $${n}_{0}^{(i)}=\frac{{x}^{(i)}-f{w}^{(i)}}{v{{\rm{\Theta }}}_{v}},$$

and thus, combining eqs (2), (5) and (7), we can express the phase difference between two cells *i* and *j* as

8 $$\begin{array}{lll}{{\rm{\Delta }}}^{(ij)}{\psi }_{n} & := & {\psi }_{n}^{(i)}-{\psi }_{n}^{(j)}={{\rm{\Delta }}}^{(ij)}{\varphi }_{0}+{\rm{\Phi }}v{{\rm{\Theta }}}_{v}[\frac{n-{n}_{0}^{(i)}}{{w}^{(i)}}-\frac{n-{n}_{0}^{(j)}}{{w}^{(j)}}]\\  & = & \{\begin{array}{ll}{{\rm{\Delta }}}^{(ij)}{\varphi }_{0}-\frac{{{\rm{\Phi }}{\rm{\Delta }}}^{(ij)}x}{{w}^{(i)}}+\frac{{{\rm{\Phi }}{\rm{\Delta }}}^{(ij)}w}{{w}^{(i)}}[f-\frac{v{{\rm{\Theta }}}_{v}}{{w}^{(j)}}(n-{n}_{0}^{(j)})] & {\rm{if}}\,{n}_{0}^{(j)}\ge {n}_{0}^{(i)}\\ {{\rm{\Delta }}}^{(ij)}{\varphi }_{0}-\frac{{{\rm{\Phi }}{\rm{\Delta }}}^{(ij)}x}{{w}^{(j)}}+\frac{{{\rm{\Phi }}{\rm{\Delta }}}^{(ij)}w}{{w}^{(j)}}[f-\frac{v{{\rm{\Theta }}}_{v}}{{w}^{(i)}}(n-{n}_{0}^{(i)})] & {\rm{if}}\,{n}_{0}^{(i)}\ge {n}_{0}^{(j)}\end{array},\end{array}$$

where in general we denote differences by

$${a}^{(i)}-{a}^{(j)}:={{\rm{\Delta }}}^{(ij)}a$$

and expanded $n-{n}_{0}^{(i)}=n-{n}_{0}^{(j)}+{n}_{0}^{(j)}-{n}_{0}^{(i)}$ for ${n}_{0}^{(j)}\ge {n}_{0}^{(i)}$, and $n-{n}_{0}^{(j)}=n-{n}_{0}^{(i)}+{n}_{0}^{(i)}-{n}_{0}^{(j)}$ for ${n}_{0}^{(i)}\ge {n}_{0}^{(j)}$.

### Special case *w*^(*i*)^ = *w*^(*j*)^ = *w*

Most coding ideas regarding phase precession implicitly assume place fields of equal width. In such a case equation (8) simplifies to

9 $${{\rm{\Delta }}}^{(ij)}{\psi }_{n}={{\rm{\Delta }}}^{(ij)}{\varphi }_{0}-\frac{{{\rm{\Phi }}{\rm{\Delta }}}^{(ij)}x}{w}.$$

A consistent phase code requires that Δ^(*ij*)^ *ψ*_*n*_ = 0 for Δ^(*ij*)^ *x* = 0, and thus the difference in phase offsets must vanish,

10 $${{\rm{\Delta }}}^{(ij)}{\varphi }_{0}=\mathrm{0,}$$

i.e., all neurons should start the place field firing at the same phase *ϕ*_0_. Therefore, when the two cells encode different positions, we have

11 $${{\rm{\Delta }}}^{(ij)}\psi =-\frac{{\rm{\Phi }}}{w}{{\rm{\Delta }}}^{(ij)}x.$$

The phase shift is therefore proportional to the place field difference consistent with the experimentally reported phase code for distance^8, 9, 32^ and illustrated in Fig. 1A.

### General case *w*^(*i*)^ ≠ *w*^(*j*)^

The most obvious difference to the special case of equal widths is that, for unequal place field widths *w* ^(*i*)^ ≠ *w* ^(*j*)^, where the phase difference depends on the cycle number *n*, the phase relation changes with time; see equation (8) and Fig. 1B. Consistency between the phase and the place code thus cannot be achieved on a cycle by cycle basis, however, the consistency argument can be generalized if one assumes that for cells with Δ^(*ij*)^ *x* = 0, the phase difference has to be zero averaged over *N* + 1 spike pairs in *N* theta cycles. Averaging is a biologically plausible computation, since spike-timing dependent synaptic learning rules^15^ that encode sequence memories are able to average over multiple repetitions of spike pairs, which in the present case would be averaging over all theta cycles in a place field traversal^4, 7, 16^.

To compute the average phase difference 〈Δ^(*ij*)^ *ψ*〉 = (*N* ^(*j*)^ + 1)^−1^∑_*n*_Δ^(*ij*)^ *ψ*_*n*_, we assume without loss of generality that *w* ^(*i*)^ > *w* ^(*j*)^, and ${n}_{0}^{(j)}\ge {n}_{0}^{(i)}$, and consequently ${n}_{0}^{(j)}+{N}^{(j)}\le {n}_{0}^{(i)}+{N}^{(i)}$, because if the narrower field (*j*) would start before or end after the wider (*i*) field, the two cells would necessarily not encode for the same position. Under these conditions we obtain

12 $$\begin{array}{rcl}\sum _{n={n}_{0}^{(j)}}^{{n}_{0}^{(j)}+{N}^{(j)}}{{\rm{\Delta }}}^{(ij)}{\psi }_{n} & = & ({N}^{(j)}+\mathrm{1)}[{{\rm{\Delta }}}^{(ij)}{\varphi }_{0}+\frac{{{\rm{\Delta }}}^{(ij)}w}{{w}^{(i)}}{\rm{\Phi }}\,f]-\frac{{{\rm{\Delta }}}^{(ij)}w}{{w}^{(i)}{w}^{(j)}}{\rm{\Phi }}\,v{{\rm{\Theta }}}_{v}\frac{{N}^{(j)}({N}^{(j)}+\mathrm{1)}}{2}\\  & = & ({N}^{(j)}+\mathrm{1)}[{{\rm{\Delta }}}^{(ij)}{\varphi }_{0}+\frac{{{\rm{\Delta }}}^{(ij)}w}{{w}^{(i)}}{\rm{\Phi }}(f-\frac{1}{2})].\end{array}$$

Consequently, the offset difference Δ^(*ij*)^ *ϕ*_0_ for which the average phase difference from equation (12) vanishes is given by

13 $${{\rm{\Delta }}}^{(ij)}{\varphi }_{0}=-\frac{{{\rm{\Phi }}{\rm{\Delta }}}^{(ij)}w}{2{w}^{(i)}}(2f-1).$$

**Figure 3:**
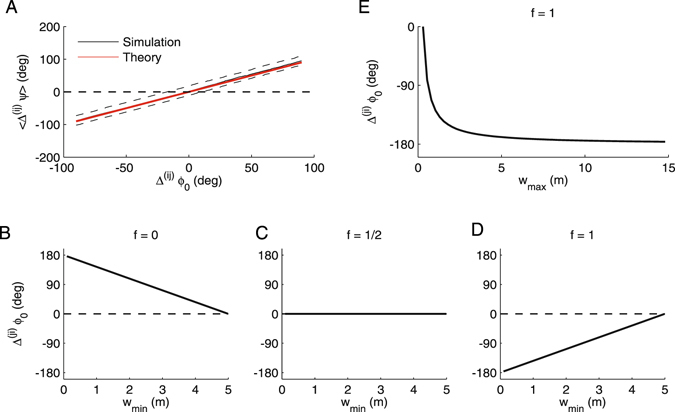
Optimal phase offsets. (**A**) To validate the theoretical results (red line) from equation (12), we derived empirical mean phase differences from simulations (see Fig. 1) of a pair of phase precessing place cells with the same center. Black line depicts the median of 50 repetitions (dashed lines are the 10- and 90-percentiles). The only free parameter was the phase range which we estimated as Φ = −360° from the simulations. In the example we used *f* = 1/2 and *v* = 0.2 m/s. (**B**–**D**) Optimal phase offset from equation (13) as a function of the width of the smaller place field, when the larger place field width was taken to be 5 m. (**E**) Optimal phase offset as a function of the larger field width while fixing the smaller field width to 30 cm.

Comparison of the average phase difference at the theoretically optimal phase offset Δ^(*ij*)^ *ϕ*_0_ from equation (13) agrees with those from a simulated pair of phase precessing cells (Fig. 3A). A biological interpretation of the fundamental equation (13) will be found in the next section.

### Biological Constraints

#### Phase Range

At first, we were asking how the theoretical phase offset Δ^(*ij*)^ *ϕ*_0_ from equation (13) fits to the observed theta wave traveling from the dorsal to the ventral pole^17–19^. In the framework of our theory a traveling wave would account for different phase offset *ϕ*_0_ at different dorso-ventral positions. The difference in phase offset Δ^(*ij*)^ *ϕ*_0_ can thus potentially be interpreted as a wave traveling from place cells of width *w* ^(*i*)^ to place cells with width *w* ^(*j*)^. We therefore computed Δ^(*ji*)^ *ϕ*_0_ = −Δ^(*ij*)^ *ϕ*_0_ for a pair of place fields as a function of the width *w﻿*_min_ of the smaller place field for changing fractions *f* that determine the reference position that the spike phase is supposed to encode the distance from (Fig. 3B–D). For *f* = 0 (spike timing encodes distance from the beginning of the field) the optimal phase offset Δ^(*ji*)^ *ϕ*_0_ for small fields is positive (delayed) corresponding to a wave traveling from large place fields to small place fields. For *f* = 1/2 (spike timing encodes distance from the center of the field) the optimal phase offset is mostly close to zero (synchronous) corresponding to a global oscillation. Finally, for *f* = 1 (spike timing encodes the distance to the end of the field) the optimal phase offset for small fields is negative (advanced) corresponding to a wave traveling from small place fields to large place fields as it would be consistent with the observed traveling direction of the theta wave. The maximum phase offset (for the smallest place fields) is the 180° (Fig. 3E) found in experiments^18, 19^.

Our work thus suggests that the theta phase offset between dorsal and ventral pole is in fact bringing the place cell spikes together in time such that neighboring cells can be encoded by downstream coincidence detector neurons irrespective of place cell width. The fundamental open question is “What are neighbors?”. This question is essentially reflected by the parameter *f*, which says that cells are neighbors if their reference positions *x* = *x*_0_ + *fw* are neighboring. Our results show that the traveling wave from dorsal to ventral pole can bring neighboring cells only into temporal coincidence for *f* = 1.

#### Traveling Waves

Mathematically the phase *φ*(*x*_*w*_, *t*) of a wave can be written as

14 $$\phi ({x}_{w},t,v)={k}_{{x}_{w},v}{x}_{w}-t{\omega }_{v}+{\alpha }_{v}$$

where ${k}_{{x}_{w},v}$ is the wave vector (2*π* divided by wavelength), *x*_*w*_ is the position along the dorso-ventral axis, *ω*_*v*_ = (2*π*)/Θ_*v*_ is the speed-dependent theta oscillation frequency, and *α*_*v*_ is some (arbitrary) constant phase. Equaling the wave phase equation (14) with the optimal phase offset Δ^(*ij*)^ *ϕ*_0_ at the ventral most pole (*w*_max_ = max_*i*_ *w*_*i*_) from equation (13) with *w* = *w* ^(*j*)^, and *f* = 1 yields

15 $${\alpha }_{v}+{k}_{{x}_{w},v}{x}_{w}={\varphi }_{0}^{(i)}-{\varphi }_{0}^{(j)}=-\frac{{\rm{\Phi }}}{2}+\frac{{\rm{\Phi }}w}{2{w}_{{\rm{\max }}}},$$

where without loss of generality we set the reference phase at the ventral pole, i.e., *φ*(*w*_max_, *t*, *v*) = −*tω*_*v*_. Since field width increases along the dorso-ventral axis, we can assume *x*_*w*_ ∝ *w*/*w*_max_ and thus obtain the optimal wave vector to be constant,

16 $${k}_{{x}_{w},v}=k.$$

The remaining term in eq. (15) can be identified with a constant phase *α*_*v*_ = −Φ/2. The wave propagation speed *c*(*v*) is the time derivative of a position *x*_*φ*_(*t*) of constant phase *φ*. Since

17 $$\phi =k{x}_{\phi }(t)-{\omega }_{v}t+\alpha ,$$

taking the time derivative yields

18 $$c(v)\equiv \frac{{\rm{d}}}{{\rm{d}}t}{x}_{\varphi }=\frac{{\omega }_{v}}{k},$$

which is a function of speed (*v*), because theta frequency (*ω*_*v*_) changes with speed. To obtain an explicit expression we assume that space *x* and place field width *w* are related by

19 $${x}_{w}=X\frac{w}{{w}_{{\rm{\max }}}},$$

where *X* quantifies the total spatial extent of the dorso-ventral axis of about 1 cm in rats^19^. Combining equations (15), (18) and (19), we obtain

20 $$c(v)=\frac{4\pi X/|{\rm{\Phi }}|}{{{\rm{\Theta }}}_{v}}.$$

For a phase range of |Φ|  = 2*π*, equation (20) yields a velocity estimate of

21 $$c(v)=\frac{2X}{{{\rm{\Theta }}}_{v}}.$$

For approximate values of *X* = 1 cm and 1/Θ_*v*_ ≈ 8 Hz this amounts to a propagation velocity of *c* ≈ 16 cm/s consistent with experiments^19^.

#### Nonlinear phase precession

The specific shape of phase precession may deviate from the linear model assumption^4, 30^. We therefore asked how much non-linear precession would affect the conclusions from the linear model. To parameterize the non-linearity we replace the linear term ${\varphi }_{0}+{\rm{\Phi }}\frac{n-{n}_{0}^{(i)}}{{N}^{(i)}}$ from eq. (2) by $({\varphi }_{0}+{\rm{\Phi }})-{\rm{\Phi }}{(1-\frac{n-{n}_{0}^{(i)}}{{N}^{(i)}})}^{\mu }$ with some positive exponent 0 < *μ* < 1. The smaller *μ* the more non-linear the phase dependence becomes. Following a similar derivation as described for the linear case we end up at an optimal phase offset of

22 $${{\rm{\Delta }}}^{ij}{\varphi }_{0}=\frac{{\rm{\Phi }}}{{N}^{(j)}+1}\sum _{m=0}^{{N}^{(j)}}[{(1-\frac{m}{{N}^{(i)}}-f\frac{{{\rm{\Delta }}}^{(ij)}w}{{w}^{(i)}})}^{\mu }-{(1-\frac{m}{{N}^{(j)}})}^{\mu }].$$

**Figure 4:**
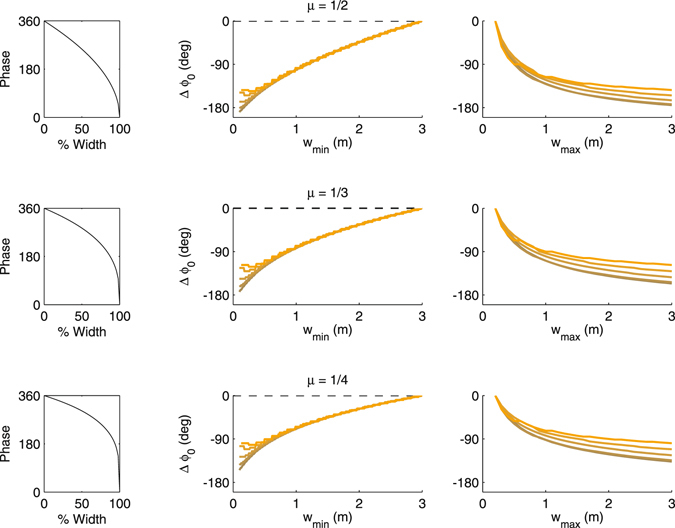
Nonlinear phase precession. (Left column) Non-linear models of phase precession with increasing curvature from top to bottom. (Middle) Optimal phase range for fixed maximal width and *f* = 1 (as in Fig. 2D). The different colors indicate different running speeds from 0.1 m/s (dark) to 1.2 m/s (bright). (Right) Optimal phase range for fixed minimal field width (as in Fig. 2E).

The optimal phase offset from eq. (22) can be numerically evaluated as shown in Fig. 4.

If the exponent *μ* is below the value of 1, we observe two main effects. First, the optimal phase offset becomes dependent on running speed *v*, however, only for large differences in place field width. Second, the optimal phase offsets stay below 180°. If the largest field width is fixed (the reference for theta phase), both speed dependence and phases below 180° can be observed for small place field widths below about 0.25 m; smaller than 180° phase offsets are found for high velocities. If, in contrast, the small field width (dorsal pole) is taken as a reference, the velocity dependence extends over a larger range of place field sizes. Also the curves are generally not linear indicating a width-dependent conduction speed of the optimal wave that is faster towards the dorsal end (corresponding to the steeper slopes).

If the non-linearity is moderate $(\mu \,\gtrapprox \,\mathrm{1/2})$ the optimal phase offsets can nevertheless still be reconciled with the observed traveling wave, particularly considering that the consistency assumption is mostly violated for high running speeds and low place field widths, where only few theta cycles contribute to potential synaptic weight changes. However, for strong non-linearities $(\mu \,\lessapprox \,\mathrm{1/4})$, the reduced phase range questions the validity of the consistency assumption. We thus conclude that the consistency assumption is quite robust for moderately non-linear phase precession and larger place field width.

## Discussion

The precise timing of hippocampal place cell firing relative to the local theta oscillation contains information about the position of an animal. Particularly it has been proposed that spatial distances are encoded by hippocampal theta phase differences. In this paper, we theoretically evaluated under which conditions this hypothesis holds. We found that despite the variable place field sizes (and hence variable precession rates), consistency between phase code and spatial distance of place fields is (approximately) possible because the hippocampal theta oscillation is associated with a traveling wave that moves from the dorsal to the ventral pole. The traveling wave thereby imposes a location-specific phase offset, which delays the spikes of the more ventral neurons in time and, in so doing, accounts for the consistency between space and phase differences on average. Our model predicts that the maximal theta phase offset between dorsal and ventral pole should be about 180 degrees as found in experiments^18, 19^.

A direct consequence of our theory is that it predicts that the reference positions to which distances are encoded by the theta phase of spikes must be the ends of the place fields, otherwise the consistency assumption could not be reconciled with the direction of the traveling wave. As a result action potentials would encode positions the animal would reach in the future and thus, this prediction is consistent with the previously proposed hypothesis that theta sequences predict future behaviors^6, 8, 10^. Rate-based theories of the hippocampal place code generally assume that place cell activity is linked to the current position of the animal. While this is a perfect assumption to optimally reconstruct animal trajectories from neural activity, our results, however, argue for the development of predictive strategies for decoding hippocampal place cell activity that optimize estimates of the future trajectory of the animal.

Although place field widths generally increase along the dorso-ventral axis, there is considerable variability at each location^21–25^, which may pose a problem to the presented theory. However, some hippocampal regions show more variability than others. For example the correlation between size and location seems the strongest in area CA3^22^, which would make this region the most likely candidate area to look at. Also the field potential oscillation is an average over the synaptic inputs of many cells and thus it cannot be excluded that individual cells at similar dorso-ventral positions show distinct intracellular theta phases. Our theory therefore predicts that for place fields with different widths but at similar dorso-ventral position (seeing the same local theta phase), the offset of the theta phase precession should be correlated with field width. Broader place fields (with shallower precession slopes) should start firing at later theta phases to ensure consistency between phase and place field distance.

Theta phase precession and spike correlations (theta sequences) have been argued to be to some degree distinct phenomena of spike time coordination^33^, i.e., theta sequences can be altered without observable changes in phase precession. This dissociation shows that the coordination of phase offsets across cells plays an important role in shaping the hippocampal ensemble code, and that the two key features, phase slope and offset, may rely on two distinct mechanisms. Particularly CA3 seems to be fundamental in coordinating the offsets, since inactivation of CA3 removes theta sequences while leaving phase precession intact on the single cell level^33^. Conversely, the phase precession slopes seem to depend at least partly on the medial entorhinal cortex (MEC) since animals with lesioned MEC displayed very little single cell phase precession^32^.
